# Fat accumulation in *Caenorhabditis elegans* triggered by the electrophilic lipid peroxidation product 4-Hydroxynonenal (4-HNE)

**DOI:** 10.18632/aging.100005

**Published:** 2008-12-18

**Authors:** Sharda P. Singh, Maciej Niemczyk, Ludwika Zimniak, Piotr Zimniak

**Affiliations:** ^1^Department of Pharmacology and Toxicology, University of Arkansas for Medical Sciences, Little Rock, AR 72205, USA; ^2^Central Arkansas Veterans Healthcare System, Little Rock, AR 72205, USA

**Keywords:** Lipid peroxidation, 4-hydroxynonenal, malonyl-CoA, acetyl-CoA carboxylase, citrate, obesity

## Abstract

Deposition and mobilization of fat in an organism are tightly controlled by multiple levels of endocrine and neuroendocrine regulation. Because these hormonal mechanisms ultimately act by affecting biochemical reactions of fat synthesis or utilization, obesity could be also modulated by altering directly the underlying lipid biochemistry. We have previously shown that genetically modified mice with an elevated level of the lipid peroxidation product 4-HNE become obese. We now demonstrate that the process is phylogenetically conserved and thus likely to be universal. In the nematode *C. elegans*, disruption of either conjugation or oxidation of 4-HNE leads to fat accumulation, whereas augmentation of 4-HNE conjugation results in a lean phenotype. Moreover, direct treatment of *C. elegans* with synthetic 4-HNE causes increased lipid storage, directly demonstrating a causative role of 4-HNE. The postulated mechanism, which involves modulation of acetyl-CoA carboxylase activity, could contribute to the triggering and maintenance of the obese phenotype on a purely metabolic level.

## Introduction

4-Hydroxynonenal (4‑HNE) is the product of peroxidation of *n*-6 polyunsaturated fatty acids [1,2]. 4‑HNE is strongly electrophilic and thus bioactive because of its potential to modify biological macromolecules, particularly proteins, via the formation of covalent adducts [3,4]. These chemical properties of 4‑HNE render the compound toxic at high concentrations [3]. However, at moderate (physiological) levels [5], 4‑HNE acts as a messenger molecule that signals the presence of a pro-oxidant state and elicits a variety of appropriate biological responses (reviewed in refs. [4, 6-9]). In this context, lipid peroxidation acts as a sensor that registers the presence of oxidants and translates it into the generation of electrophiles such as 4‑HNE. Compared with oxidantssuch as most types of ROS (reactive oxygen species), 4‑HNE is chemically better suited for the role of a signaling molecule because of its longer half-life and thus greater range of diffusion, and a higher selectivity for reaction with specific targets.

Multiple physiological and pathophysiological situations can result in oxidative stress. Among them is excessive fat accumulation that leads to mild but chronic inflammation characterized by focal necrosis and recruitment of macrophages to the adipose tissue; inflammation is both local and systemic [10-12]. The oxidative stress that accompanies inflammation would be expected to trigger lipid peroxidation and 4‑HNE production. Indeed, higher levels of 4‑HNE have been found in obese than in non-obese humans and mice [13,14].

Several biologically fundamental target processes responsive to 4‑HNE signaling have been identified, including modulation of apoptosis, activation of stress response pathways, cell proliferation and differentiation, mitochondrial coupling, and others (reviewed in refs. [9, 15-18]). Results presented in this paper, together with our previous data [19], define a new target of 4‑HNE signaling: adipogenesis. This would place 4‑HNE not only downstream [14] but also upstream of fat accumulation, resulting in a positive feedback loop. In this paper, we present evidence for a biochemical mechanism by which 4‑HNE could trigger increased lipid storage, and discuss possible adaptive and maladaptive roles of the self-reinforcing loop that involves triglycerides and 4‑HNE, in particular, in the context of aging.

## Results

**Expression of the *gst‑10* gene product, which is capable of conjugating 4‑HNE, is inversely related to the level of whole-body 4‑HNE-protein adducts**

**Figure 1.**
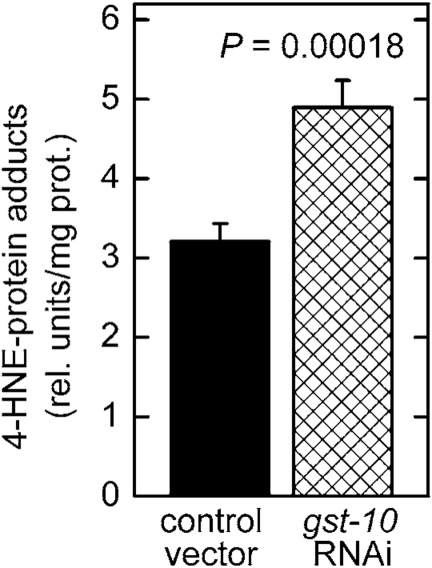
Increased level of 4‑HNE-protein adducts in *C. elegans* with silenced *gst‑10* gene. RNAi silencing of *gst‑10* was carried out as previously described [21] in *C. elegans* strain Bristol-N2; worms fed bacteria transformed with an insert-free L4440 feeding vector served as the control. The level of 4‑HNE-protein adducts was determined in 5-day-old worms by competitive ELISA [93]. The values shown are means ± S.D. of 4 biological replicates (independently grown batches of worms) per group.

As we have previously shown [20,21], the capacity of *C. elegans* to conjugate 4‑HNE with glutathione was reduced by RNAi-mediated knockdown of CeGSTP2-2, the product of the *gst‑10* gene. The loss of 4‑HNE-conjugating activity was only partial because of the existence of multiple GSTs capable of conjugating 4‑HNE [20]. Nevertheless, silencing of *gst‑10* resulted in a greater than 50% increase of the level of whole-body 4‑HNE-protein adducts (Figure 1). Conversely, overexpression of *gst‑10* results in an enhanced capacity to conjugate 4‑HNE and in a decrease in the content of 4‑HNE-protein adducts [22]. Because the biological effects of 4‑HNE are thought to be mediated mostly by formation of covalent adducts with proteins, a process that frequently alters protein function (reviewed in refs. [4, 6-9]), the observed changes in adduct amounts could be functionally significant.

## Fat accumulation in

**Figure 2.**
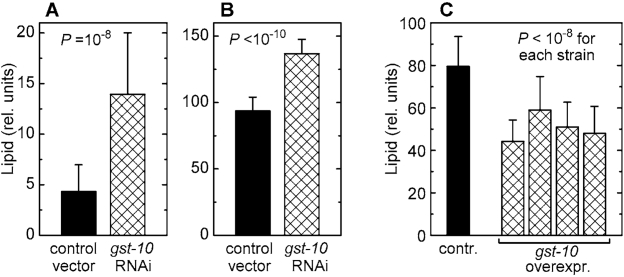
Lipid content of *C. elegans* with altered expression of *gst‑10*. **(A)** and **(B)**: worms fed bacteria carrying the insert-free L4440 feeding vector (control) and worms subjected to RNAi against *gst‑10*. Lipid content was measured by Nile red fluorescence **(A)** and by Sudan black staining **(B)**. **(C)**: lipid content (by Sudan black) in worms transformed with the insert-free pPD49.26 vector (control) and in four transgenic *C. elegans* lines that overexpress *gst‑10*. Bars represent means ± S.D. (n = 50 worms per group) of pixel intensities integrated on worm micrographs.

Worms subjected to RNAi against *gst‑10* accumulate significant amounts of fat (Figures 2A and 2B). The use of two independent lipid stains, Nile red [23,24] and Sudan black [25], minimizes the likelihood of artifacts. In contrast to animals subjected to RNAi, several *C. elegans* lines overexpressing the *gst‑10* gene product [22] had a lean phenotype (Figure 2C). This lean phenotype was confirmed using a second, independently derived control line carrying an insert-free vector (data not shown), thus establishing that the low fat content of *gst-10*-overecxpressing worms was not due to an aberrant control line. The data demonstrate an inverse proportionality between *gst‑10*-linked 4‑HNE-conjugating activity and fat accumulation in *C. elegans*.

## Disruption of oxidative metabolism of 4‑hne leads to a fat accumulation phenotype resembling that seen in 4‑hne conjugation-defective

**Figure 3.**
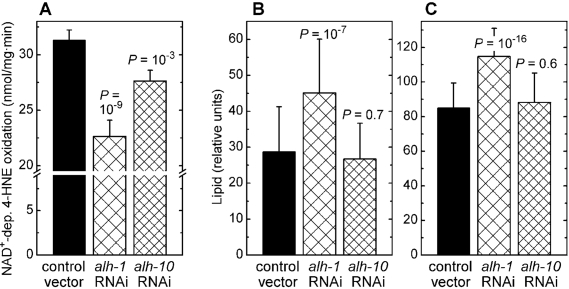
Effects of RNAi against aldehyde dehydro-genases *alh-1* and *alh-10*. **(A)** Effect of RNAi against *alh-1* and *alh-10* on NAD^+^-dependent 4‑HNE oxidation capacity of worm homogenates. **(B)** and **(C)**: Fat content in *C. elegans* subjected to RNAi against *alh-1* and *alh-10*, determined by Nile red **(A)** and Sudan black **(B)** staining followed by pixel integration. Means ± S.D. (n = 51 worms per group) are shown. In all experiments, statistical significance versus insert-free L4440 feeding vector control was assessed by the *t*‑test with Bonferroni correction.

In addition to glutathione conjugation, 4‑HNE can undergo reductive or oxidative metabolism (reviewed in refs. [5, 6]). In mammalian cells, several aldehyde dehydrogenases that catalyze the oxidation of the carbonyl group of 4‑HNE are physiologically important [2,26,27]. Of the twelve aldehyde dehydrogenase (*alh*) genes of *C. elegans* (WormBase release WS194), we investigated *alh-1* which has a high, and *alh-10* which has a moderate contribution to whole-body NAD^+^-dependent 4‑HNE oxidation (Figure 3A). Silencing of the *alh-1* gene led to a statistically significant increase in fat accumulation, as measured either by Nile red (Figure 3B) or by Sudan black (Figure 3C) staining. In contrast, RNAi against *alh-10* had no effect on the level of stored lipids (Figure 3B and C). These results show that the inverse relationship between the accumulation of fat and the organism's capacity to metabolize 4‑HNE is not limited to glutathione conjugation of 4‑HNE by the *gst‑10* gene product, but that it extends to another type of 4‑HNE metabolism catalyzed by an unrelated enzyme.

**Direct exposure of *C. elegans* to synthetic 4‑HNE leads to an increase of 4‑HNE-protein adducts and causes the fat accumulation phenotype**

**Figure 4.**
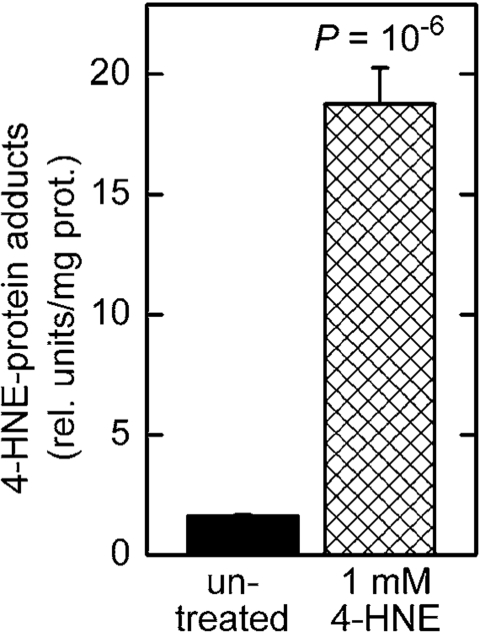
Increased level of 4‑HNE-protein adducts in *C. elegans* treated with 4‑HNE. Synchronized L4 worms were kept in liquid culture for 3 days in medium containing 1 mM 4‑HNE. The medium was replaced every 12 hr with fresh medium containing 4‑HNE. The level of 4‑HNE-protein adducts was determined in 5-day-old worms by competitive ELISA [93]. The values shown are means ± S.D. of 4 biological replicates (independently grown batches of worms) per group.

Treatment of *C. elegans* in liquid culture with chemically synthesized 4‑HNE caused an increase in the level of 4‑HNE-protein adducts (Figure 4). This increase was greater than that seen in worms subjected to RNAi against *gst‑10* (Figure 1), possibly because *gst-10* expression is restricted to relatively few cells of *C. elegans* [22]. Given the limitations on long-range diffusion of 4‑HNE [28], the effects on 4‑HNE that result from *gst-10* disruption may also be local, and would appear as moderate when averaged over the entire organism. In contrast, most or all tissues are likely to be affected in worms exposed to 4‑HNE-containing medium. In addition to the tissue distribution of 4‑HNE, the relatively high concentration (1 mM) of the compound in the growth medium could have also contributed to the pronounced increase in adducts. However, it is important to note that the biologically active concentration of 4‑HNE in tissues of *C. elegans* is approximately three orders of magnitude lower than the concentration of externally added 4‑HNE [20]. Thus, the effective 4‑HNE concentration was likely on the order of 1 μM, well within a physiologically achievable range (low micromolar; 5).

**Figure 5.**
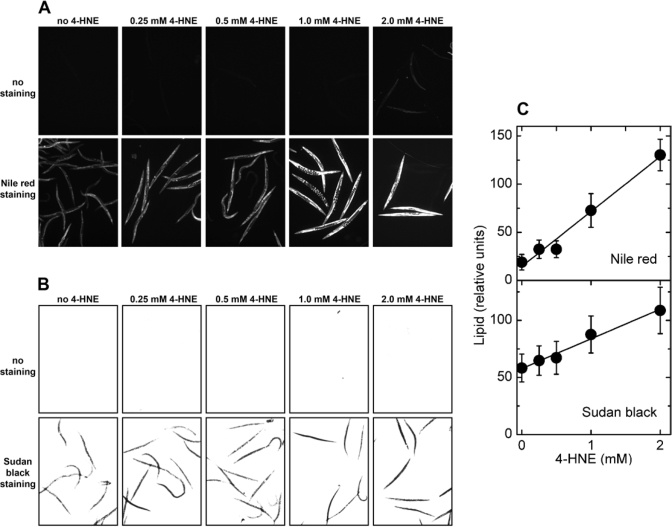
Effects of direct treatment with 4‑HNE on lipid accumulation in *C. elegans*. Synchronized L4 worms were kept in liquid culture for 3 days in medium containing the indicated concentration of 4‑HNE. The medium was replaced every 12 hr with fresh medium containing 4‑HNE. **(A)** fluorescence of worms treated with the indicated concentration of 4‑HNE but not stained (*upper row of images*), and animals exposed to 4‑HNE and stained with Nile red (*lower row of images*). **(B)** brightfield images of worms treated with the indicated concentration of 4‑HNE and directly photographed (*upper row of images*), or photographed after staining with Sudan black (*lower row of images*). **(C)**: for each 4‑HNE concentration, lipid content was measured in three independently treated worm populations by quantitation of Nile red fluorescence (*upper plot*) or Sudan black staining (*lower plot*).

Exposure of *C. elegans* to synthetic 4‑HNE led to storage of excess lipids that increased with increasing medium concentration of 4‑HNE, as shown by either Nile red or Sudan black staining (Figure 5A and B, respectively). No significant fluorescence was visible in the absence of Nile red (Figure 5A, upper row of images), demonstrating that the observed signal is not caused by increased autofluorescence in response to the treatment with 4‑HNE. This is important because 4‑HNE has been implicated in the process of lipofuscin formation [29]. Similarly, there was no signal in brightfield images of worms treated with 4‑HNE but not stained (Figure 5B, upper row), whereas identically treated animals that were stained with Sudan black yielded a strong signal under the same exposure conditions (Figure 5B, lower row of images). Quantitation of multiple images shows that the amount of accumulated fat was linearly proportional to the medium concentration of 4‑HNE in the range of 0.25 - 2 mM (Figure 5C).

**Fat accumulation triggered by 4‑HNE is accompanied by metabolic changes consistent with increased *de novo* synthesis and/or decreased β-oxidation of fatty acids**

**Figure 6.**
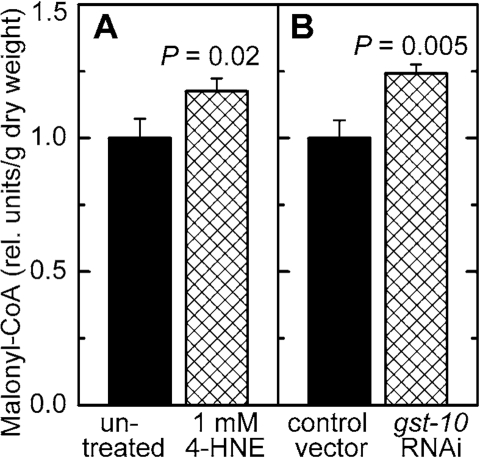
Effect of increased 4‑HNE levels on malonyl-CoA concentration. *C. elegans* were either treated with 1 mM 4‑HNE according to the protocol described in the legend of Figure 5 **(A)** or subjected to RNAi to *gst‑10* **(B)**). The results were normalized to the respective control and are shown as mean ± S.D. of 3 independently grown worm populations. In **(A)**, the control (*C. elegans* Bristol-N2 in liquid culture in the presence of *E. coli* OP50 but without 4‑HNE) contained 16.5 ± 1.2 ng malonyl-CoA/mg dry weight; in **(B)**, the level of malonyl-CoA in the control (*C. elegans* Bristol-N2 grown on plates seeded with *E. coli* HT115(DE3) transformed with insert-free L4440 feeding vector) was 12.2 ± 0.8 ng/mg dry weight.

**Figure 7.**
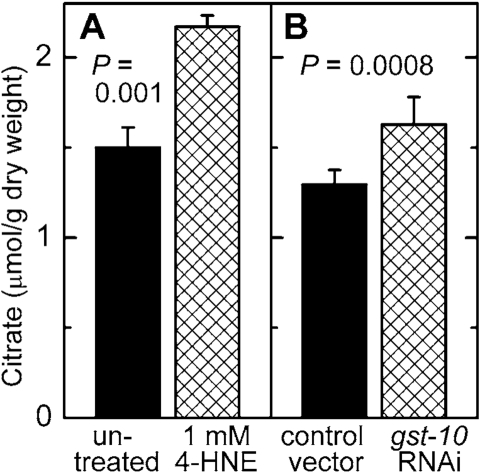
Effect of increased 4 HNE levels on citrate concentration. C. elegans were either treated with 1 mM 4- HNE according to the protocol described in the legend of Figure 5 **(A)** or subjected to RNAi to gst 10 **(B)**. The results are shown as means ± S.D. of 3 to 6 independently grown worm populations.

Malonyl-CoA is a metabolite of key importance in lipid homeostasis (see Discussion). Malonyl-CoA was measured in *C. elegans* in which the level of 4‑HNE was increased by either of two methods: direct treatment with 4‑HNE, or RNAi against *gst‑10* which leads to an impairment of 4‑HNE removal. In both situations, the concentration of malonyl-CoA was elevated (Figure 6A and B). Worms treated in the same way were also assayed for citrate, a precursor and activator of malonyl-CoA synthesis (see Discussion). In analogy to malonyl-CoA, the concentration of citrate was increased as a consequence of diminished metabolic elimination of 4‑HNE or in response to direct supplementation of worms with 4‑HNE (Figure 7).

## Discussion

Experimental manipulation of enzymes that catalyze elimination of 4‑HNE from tissues modulates the steady-state level of 4‑HNE and affects the amount of stored fat in *C. elegans*. Specifically, inhibition of either conjugation or oxidation of 4‑HNE leads to increased fat accumulation, whereas overexpression of a 4‑HNE-conjugating enzyme results in a lean phenotype (Figure 2 and Figure 3). These observations are consistent with the obese phenotype we have previously reported for *mGsta4* null mice which have an impaired ability to conjugate 4‑HNE [19], indicating that the link between abundance of 4‑HNE and fat storage is conserved between evolutionarily distant species.

We propose that 4‑HNE, rather than another substrate or function of the enzymes that are being experimentally manipulated, is a causative factor in the enhanced storage of lipid. We favor this model for the following reasons:(i) Except for 4‑HNE, there is no known common substrate of the two rather dissimilar *C. elegans* enzymes used in the present work, *i.e.*, the products of *gst‑10* and *alh‑1* genes. Moreover, 4‑HNE is the only known common substrate of the above-mentioned two enzymes and the murine mGSTA4-4 [30].

(ii) Fat deposition was reported for yeast directly treated with 4‑HNE [31], an experimental design that points to 4‑HNE as the causative agent.

(iii) *mGsta4* null mice in the 129/sv genetic background have an elevated tissue levels of 4‑HNE and are obese, while *mGsta4* null mice in C57BL genetic background have near-normal levels of 4‑HNE and are not obese [19]. The proportionality of 4‑HNE and fat deposition in otherwise closely related systems suggests a causal role of 4‑HNE in lipid accumulation in the murine model.

(iv) Overexpression of murine aldose reductase Akr1b7 [an enzyme catalyzing reductive metabolism of 4-HNE, ref. 32] inhibits adipogenesis, and disruption of the same enzyme accelerates adipogenesis [33]. Concordant results obtained with mGSTA4-4 and Akr1b7 indicate that a common substrate, such as 4‑HNE, is responsible for the phenotype.

While alternative explanations could be invoked for each of the findings listed above, a strong case for a causal involvement of 4‑HNE can be made if the arguments are considered in aggregate. It is unlikely that several enzymes that act on distinct functional groups of 4‑HNE would all share a hypothetical additional substrate. The reactions these enzymes catalyze include Michael addition of a nucleophile to a polarized double bond (GSTs), NAD^+^-dependent oxidation of an aldehyde to a carboxylic acid (aldehyde dehydrogenase), and NAD(P)H-dependent reduction of an aldehyde to an alcohol (aldose reductase). To be able to undergo all three reactions, a substrate must contain the requisite target functional groups; this requirement restricts the set of possible substrates to α,β-unsaturated carbonyl compounds such as 4‑HNE. Moreover, experimental interventions that lower the expression of 4‑HNE-conjugating GSTs in *C. elegans* (*gst-10*) and in mice (mGSTA4-4) resulted in a similar phenotype, *i.e.*, increased fat storage. The two enzymes are not orthologs, and their ability to conjugate 4‑HNE probably evolved independently [22,34]. It is unlikely that both enzymes acquired by chance another shared activity or function that is unrelated to conjugation of 4‑HNE, indicating that the parallel effects of disruptingmGSTA4-4 and *gst-10* are indeed mediated by 4‑HNE.

As outlined above, experimental modulation of 4‑HNE-metabolizing enzymes indicates that 4‑HNE can trigger fat accumulation. However, even though highly persuasive, the evidence is indirect. The existence of an alternative substrate of the three 4‑HNE-metabolizing enzymes is unlikely but cannot be ruled out with certainty. In addition, decreasing the expression of the three enzymes could lead to lipid accumulation via three independent mechanisms, none of them involving 4‑HNE - a hypothesis that is implausible but not impossible. Therefore, we wished to test a possible causative role of 4‑HNE without relying on enzymes. For this purpose, we took advantage of the unique experimental opportunities afforded by *C. elegans*, a multicellular animal that can be handled by methods developed for microorganisms or cultured cells [35]. This includes the option to grow *C. elegans* in liquid medium that can be supplemented with compounds to be tested. Exposure of *C. elegans* to synthetic 4‑HNE led to dose-dependent lipid deposition (Figure 5) similar to that triggered by silencing of either *gst-10* or *alh-1*. While this finding does not formally rule out the possibility that hypothetical alternative substrates of *gst-10* and *alh-1* are also involved, it proves that 4‑HNE is sufficient to elicit the fat accumulation phenotype.

The increase in stored fat was similar for worms directly exposed to 4‑HNE (Figure 5) and for those with silenced 4‑HNE-metabolizing enzymes (Figure 2 and Figure 3), even though the former treatment was markedly more effective in raising the level of 4‑HNE-protein adducts (Figure 4 versus Figure 1). These findings could indicate that silencing *gst‑10* or *alh-1* raises the concentration of 4‑HNE in selected tissues, including those involved in regulating fat storage. In contrast, exposure of animals to external 4‑HNE is likely to affect the entire body. Either treatment would reach the critical target tissues and trigger fat accretion, but exposure to 4‑HNE in the medium would generate more total 4‑HNE adducts. Further work is needed to identify the cells or tissues that are requires for increased fat storage in response to 4‑HNE, although head neurons are a possibility. Head neurons express the *gst‑10* [22] as well as the *alh-1* gene products (WormBase release WS194).

What is the molecular mechanism by which 4‑HNE increases the level of stored fat? A clue is offered by our finding that the concentration of malonyl-CoA was increased in *C. elegans* with disrupted enzymatic removal of 4‑HNE, as well as in animals directly exposed to 4‑HNE (Figure 6). Malonyl-CoA has a dual metabolic function: it is the substrate for fatty acid synthesis, and it prevents fatty acid β-oxidation [reviewed in ref. 36]. Therefore, elevated malonyl-CoA levels lead to an accumulation of fatty acids and, consequently, of triglycerides. In fact, experimental lowering of malonyl-CoA levels results in a lean phenotype in mammals [37-39] as well as in *C. elegans* [40]. Conversely, liver-specific disruption of AMPK, expected to increase malonyl-CoA levels, results in elevated hepatic lipogenesis [41,42]. These data indicate that the higher level of malonyl-CoA that we observed in worms with increased 4‑HNE could account for the concomitant fat accumulation.

Malonyl-CoA is formed from acetyl-CoA by the enzyme acetyl-CoA carboxylase (ACC). Although the steady-state tissue concentration of malonyl-CoA depends on the rate of its synthesis by ACC as well as its removal rate by fatty acid synthase or by malonyl-CoA decarboxylase, the activity of ACC is considered to be the major physiological determinant of malonyl-CoA levels at least in some tissues [43]. Therefore, the regulation of ACC activity is of central importance for fat homeostasis. Not surprisingly, ACC has been proposed as a target for pharmacological intervention [39,44].

Regulation of ACC is mostly post-translational. In mammals, a major regulatory mode is phosphorylation, particularly by AMPK [45,46]. Although *C. elegans* ACC lacks the canonical target sequence for AMPK phosphorylation [47], worm ACC is probably still regulated by AMPK, perhaps using an alternative phosphorylation site, because AMPK is required for metabolic modulation of fat stores [48]. Further work will be needed to elucidate the details of ACC phosphorylation in *C. elegans*.

**Figure 8.**
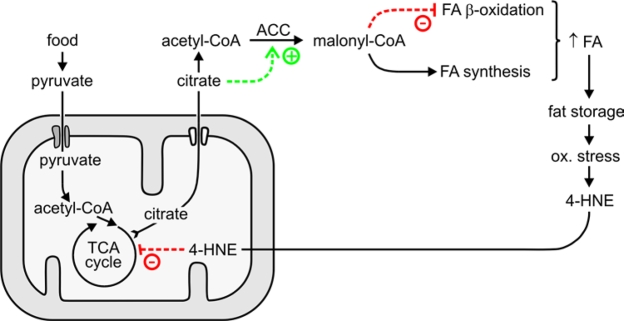
Proposed role of 4‑HNE in the generation and maintenance of fat stores.

In addition to phosphorylation, ACC is acutely regulated by allosteric effectors including citrate (reviewed in refs. [47, 49]). In the well-fed state, excess citrate is withdrawn from the TCA cycle and transported out of mitochondria. In the cytosol, citrate has a dual function in fat synthesis: (i) it allosterically activates ACC, and (ii) it is converted by citrate lyase to acetyl-CoA, the substrate of ACC (Figure 8). The resulting higher flux through ACC increases the supply of malonyl-CoA.

A partial inhibition of the tricarboxylic acid cycle enzyme aconitase lead to increased steady-state levels of citrate [50]. This is relevant to the present work because aconitase is susceptible to inhibition by 4‑HNE. It has been reported that adducts of 4‑HNE with aconitase form when isolated mitochondria are exposed to 4‑HNE [ref. 51 and references therein], and that treatment of cultured cells with 4‑HNE leads to a decrease in aconitase activity [52]. We found that *mGsta4* knockout mice have lower aconitase activity and higher citrate levels than wild-type animals [19]. We now demonstrate that increasing the levels of 4‑HNE, either by direct treatment with the compound or by disruption of the metabolic disposal of 4‑HNE, results in an elevated citrate concentration in *C. elegans* (Figure 7). Thus, our results support a model in which 4‑HNE partially inhibits aconitase, causing an increase in the steady-state concentration of citrate. The latter metabolite augments the flux through ACC by providing both the substrate and the allosteric activator of the enzyme. This leads to more malonyl-CoA production, and thus to more fatty acid synthesis and less fatty acid β-oxidation. The accumulating fatty acids are converted to storage fat. The model is schematically depicted in Figure 8.

We propose that the chain of events leading from 4‑HNE to increased fat storage operates not only when 4‑HNE levels are experimentally perturbed, but that it is part of normal physiology. In the wild, access to food is typically intermittent. It is therefore highly adaptive to maximize stores of metabolic fuel when food is available. Such stores increase the probability of surviving "lean" periods. Feeding triggers the synthesis and deposition of fat. At least in mammals, increased fat storage correlates with a more pronounced pro-inflammatory and pro-oxidative state [53, 54]. Oxidative stress is expected to facilitate lipid peroxidation and raise 4‑HNE levels. In fact, it has been shown directly that feeding-induced obesity in mice causes an increase in tissue concentration of 4‑HNE [14]. In turn, elevated 4‑HNE realigns metabolism to favor fat deposition by increasing malonyl-CoA production, as described by us in the present report. We propose that these two partial processes - fat-induced 4‑HNE generation and 4‑HNE-triggered fat accumulation - form a positive feedback loop that perpetuates lipid storage as long as food is available. The proposed self-reinforcing loop is schematically depicted in Figure 8.

Although self-sustaining while food is available, the postulated regulatory circuit involving 4‑HNE is not irreversible. When food becomes scarce (*i.e.*, under caloric restriction conditions), metabolic and sensory inputs would act to shift metabolism to a mode that emphasizes somatic maintenance and utilization rather than storage of fat [55-57]. In this physiologic state, ROS generation is decreased, perhaps because of altered mitochondrial function [58]. Less ROS translates into less 4‑HNE production. At the same time, decreased insulin-like signaling would augment the Daf-16-dependent expression of the *gst‑10* gene that encodes a 4‑HNE-conjugating enzyme [21,22]. The combination of limited supply and enhanced removal would lower the concentration of 4‑HNE, thus decreasing the production of malonyl-CoA by reversing the mechanisms summarized in Figure 8. This would, in turn, limit fatty acid synthesis and permit fatty acid β-oxidation, as required for the utilization of fat as a metabolic fuel.

The mechanism proposed in Figure 8 suggests that the metabolic "pro-adipose bias" observed in the well-fed state can be reversed not only by withdrawal of food, but also by withdrawal of 4‑HNE, even in the continuing presence of excess food. Pharmacological agents that act as 4‑HNE scavengers have been reported [59-64]. Clearly, the 4‑HNE-mediated mechanism we propose is not the only process that favors fat accumulation. Nevertheless, the potential importance of modulating 4‑HNE levels in preventing or treating obesity could be considerable.

Modulation of *gst‑10* expression has opposite effects on fat accumulation and on life span of *C. elegans*. Worms with silenced *gst‑10* (and elevated 4‑HNE) accumulate fat (Figure 2) and have a shortened life span [21], whereas overexpression of *gst‑10* results in a lean phenotype (Figure 2) and it prolongs life [22]. In mammals, on an epidemiological level obesity correlates with an increased risk for metabolic syndrome and earlier mortality [reviewed in ref. 65]; it has been recently suggested that oxidative stress-triggered fat deposition not only correlates with, but causes aging [66]. However, the link between fat accumulation, disease, and longevity, while undeniable, is complex and defies simple generalizations. The tissue distribution of lipid is an important factor. Recent data indicate that the primary trigger of metabolic syndrome in mammals is not total but ectopic fat, and that fat in adipose tissue may play a lesser role or could actually be protective by preventing the accretion of ectopic fat [65,67-71]. Another factor that is relevant to longevity is the chemical nature of lipids, in particular the degree of unsaturation of fatty acids and the position of double bonds; highly peroxidizable polyunsaturated fatty acids may limit life span [72-77]. In *C. elegans*, fat accumulation has been found to be associated with either shortened or extended life span [48,78-82], probably reflecting differences in fat localization, structure, or timing of fat abundance in the course of development. Recent work has demonstrated that lipid stores provide a metabolic link in the signaling pathway from germline stem cells to longevity, as well as in the mechanism by which lower Daf-2 activity leads to an extension of life [83]. In the latter work, the decreased fat stores that were associated with a longer life span were caused by induction of a triglyceride lipase. We report the same inverse relationship between fat and longevity when lipid stores are modulated by altering the supply of fatty acids [this work and refs. 20-22]. Therefore, our data support the notion that accumulation of fat, whether through increased synthesis or lowered utilization, can shorten life span of *C. elegans*.

In summary, we have identified a biochemical mechanism, mediated through the lipid peroxidation product 4‑HNE, that promotes fat accumulation. The mechanism is part of a positive feedback loop that confers a pro-adipose bias on organismal lipid homeostasis. The proposed mechanism differs from the better-known endocrine regulation of fat-related physiology by its purely biochemical nature. Therefore, the mechanism may offer heretofore unexplored opportunities for intervention.

## Materials and methods

*C. elegans* culture conditions. *C. elegans* Bristol-N2 were cultured at 20°C on 2% agar plates containing nematode growth medium (NG medium: 25 mM potassium phosphate, pH 6.0, 50 mM NaCl, 0.6% (w/v) peptone, 5 μg/ml of cholesterol, 1 mM MgSO_4_, 1 mM CaCl_2_) and seeded with *Escherichia coli* strain OP50 [modified from ref. 84]. *E. coli* OP50 were grown in LB medium (10 g/l of Bacto-Tryptone, 5 g/l of Yeast Extract, 10 g/l of NaCl, pH 7.0).

The generation and maintenance of transgenic *C. elegans* overexpressing *gst‑10* was described previously [22]. In experiments employing RNAi, *C. elegans* Bristol-N2 worms were fed *E. coli* strain HT115(DE3) transformed with an insert-free L4440 feeding vector (control), or the same vector carrying the appropriate insert. Specifically, RNAi against *gst‑10* was conducted according to [21]. RNAi against *alh-1* and *alh-10* was carried out using clones III-2N01 and X-3B18, respectively, from the RNAi library [85,86] purchased from Geneservice Ltd, Cambridge, U.K. To induce the expression of double-stranded RNA, bacteria were treated with 1 mM isopropyl-β-d-thiogalacto-pyranoside (IPTG).

For liquid culture, synchronized *C. elegans* at the L4 stage (2.5 days post-hatch) were rinsed off plates and approximately 1,500 worms were placed in 3 ml S-buffer [0.1 M NaCl, 0.05 M potassium phosphate, pH 6.0; ref. 84] supplemented with cholesterol (3 μl of a 5 mg/ml stock solution in ethanol) and with the same *E. coli* strain that was used for the initial growth on plates at a final density of A_600_= 1.0. The cultures were maintained at 20°C except for *gst-10* overexpressors which were grown at 25°C. The medium was replaced every 12 hours with fresh medium containing the components listed above, including fresh bacteria.

Treatment of *C. elegans* with 4‑HNE. Worms were maintained in liquid culture for 3 days under the conditions described above, except that 4‑HNE (at concentrations ranging from zero to 2.0 mM) was added to the growth medium. Therefore, worms were exposed to fresh 4‑HNE every 12 hours for a total of three days.

Nile red and Sudan black staining of *C. elegans.* For Nile red staining, worms were grown in liquid culture in medium supplemented with 75 ng/ml Nile Red (added as a 0.5 mg/ml stock solution in acetone). After 3 days of treatment with Nile red and, where used, with concurrently added 4‑HNE, worms were washed for 3 × 10 min with S-buffer, and were placed on a slide and covered with a coverslip. Randomly selected fields were photographed under a fluorescence microscope (Nikon Eclipse E1000 equipped with a digital camera) using a 4× objective and an identical exposure time (300 ms) for all images. The filter cube consisted of a 465-495 nm excitation filter, a 505 nm long pass dichroic mirror, and a 540 nm long pass emission filter. Fluorescence spectra of Nile red-stained lipids of different polarity [23,24,87] indicate that this filter combination registers both neutral and polar lipids, although excitation for polar lipids is suboptimal. Therefore, under the conditions used, mostly neutral fat is quantitated.

For Sudan black B staining [88], worms were synchronized and processed as described in the preceding paragraph, except that Nile red was omitted from the solutions. After three days in liquid culture, worms were washed in S-buffer for 30 min, fixed with 1% paraformaldehyde in S buffer, and subjected to three freeze-thaw cycles. The animals were then dehydrated through consecutive washes with 25%, 50%, and 70% ethanol. Staining was performed overnight (approximately 16 hours) in a 50% saturated solution of Sudan black B in 70% ethanol. Following staining, worms were washed for 4 × 10 min with M9 buffer [6 g Na_2_HPO_4_, 3 g KH_2_PO_4_, 5 g NaCl and 0.25 g MgSO_4_·7H_2_O per liter; ref. 84] and randomly chosen fields were photographed under a brightfield microscope (Nikon Eclipse E1000 equipped with a digital camera) with Nomarski optics and a 4× objective. The exposure time (10 ms) was identical for all images.

All images were saved as monochrome 16-bit TIFF files, and were analyzed in the ImageJ program http://rsbweb.nih.gov/ij. Files were converted to 8-bit depth, were inverted for Sudan black stains, and the perimeter of 50 to 100 non-overlapping, complete worm images was traced manually. The mean pixel intensity was recorded for each scored animal after background subtraction. The mean intensity represents the amount of dye, and thus amount of lipid, per unit of surface area in the image, *i.e.*, the density of lipid in the organism. This lipid density parameter corresponds to that used by Ashrafi et al. [40], except that it is averaged over the entire organism rather than over a chosen region of the worm body. We also calculated a related parameter, the total amount of fat in an organism, by multiplying the mean pixel intensity by the number of pixels in the image of each worm. Analysis of the total lipid content (not shown) and of the lipid density presented in the results section yielded the same conclusions.

Enzyme assays **.** Conjugation of 4‑HNE with gluta-thione (GST activity) was measured according to Alin et al. [89] in whole-body homogenates of *C. elegans* obtained as described previously [20]. For the determination of NAD^+^-dependent oxidation of 4‑HNE (aldehyde dehydrogenase activity), worms from four 100-mm plates (approximately 10,000 animals) were washed with 50 mM Tris-HCl, pH 7.4, 5 mM EDTA, and stored at -70°C. Immediately before use, 0.1 ml of the above buffer were added to the worm pellet, and the suspension was sonicated on ice for 3 × 10 s and centrifuged for 20 min at 12,000 *g* at 4°C. Aldehyde dehydrogenase activity was assayed in the supernatant according to [90], except that 1 mM 4‑HNE was used as the substrate instead of benzaldehyde, and 1 mM NAD^+^ was used as the co-substrate instead of NADP^+^.

Determination of metabolite levels. For the determination of citrate, frozen worms were homogenized in 1 M perchloric acid. Following centrifugation, the supernatant was neutralized and citrate was measured by a coupled enzyme assay utilizing citrate lyase and malate and lactate dehydrogenases [91], as implemented in the citric acid determination kit by Boehringer Mannheim/ R-Biopharm (Roche, Marshall, MI).

To assay malonyl-CoA, worm pellets were frozen and stored at -70°C. On the day of assay, the pellets were pulverized in a mortar at liquid nitrogen temperature, and were lyophilized overnight. The weight of the powder was recorded, and the samples were processed and malonyl-CoA was determined by HPLC by a modification of the method of Lazzarino et al*.*[92] as described by us previously [19].

Assay of 4‑HNE-protein adducts. The level of 4‑HNE-modified proteins was determined by competitive ELISA [93] as modified by us previously [20].

Materials. 4‑HNE dimethylacetal was synthesized according to [94,95]. 4‑HNE was prepared on the day of use by hydrolysis of 4‑HNE dimethylacetal in 1 mM HCl for 1 hour at room temperature. Sudan black B was from Aldrich (Milwaukee, WI; catalog number 19,966-4), and Nile red was from Molecular Probes (Carlsbad, CA; catalog number N-1142).
